# Rechallenge to Carboplatin in Children with Low Grade Glioma and Carboplatin Hypersensitivity Reactions

**DOI:** 10.3389/fphar.2017.00179

**Published:** 2017-04-07

**Authors:** Antonio Ruggiero, Daniela Rizzo, Martina Catalano, Palma Maurizi, Stefano Mastrangelo, Giorgio Attinà, Riccardo Riccardi

**Affiliations:** Division of Pediatric Oncology, Catholic University of RomeRome, Italy

**Keywords:** carboplatin, hypersensitivity, re-exposition, children, low grade glioma

## Abstract

**Background:** Carboplatin based regimens have demonstrated activity in pediatric patients with low grade gliomas (LGG). However, carboplatin hypersensitivity reactions (CHRs) may be a major problem leading to premature cessation of an effective therapy. The objectives of this study were to describe the prevalence, characteristics and management of CHR.

**Methods:** We performed a retrospective review of children with LGG treated between January 1994 and July 2015 with carboplatin and vincristine who had a documented CHR. We identified two groups: the first was treated following the schema proposed by Packer et al., and re-exposed to carboplatin using a desensitization protocol; the second was treated according to protocol SIOP LGG 2004 and re-exposed with the infusion time prolonged.

**Results:** CHRs were observed in 16 patients (34%) out of 47. Hypersensitivity reactions occurred in 6 patients (20.7%) of the first, and 10 patients (55.5%) of the second group, respectively. The grade 2 reactions were the most common. The median number of carboplatin doses administered at the first episode of CHR was 7 (range, 3–9) for the first group, and 8.5 (range, 5–11) for the second, respectively. Six patients were re-exposed to carboplatin using a desensitization protocol; 10 with a prolonged infusion time. Overall success rate for re-exposition was 43.75% (100% and 10%, respectively) (*P* = 0.001).

**Conclusions:** Our results show that re-exposure is a safe alternative to abandoning carboplatin. Desensitization showed greater effectiveness compared to a prolonged infusion time, which allowed the patients to receive effective treatment without adverse reactions.

## Introduction

Low-grade glial tumors are the most common brain tumors of childhood (Packer et al., 2008). The primary treatment modality is surgery while chemotherapy and radiotherapy have been reserved for recurrent and progressive cases with deterioration of visual or pituitary function (Trisciuzzi et al., 2004). Carboplatin, usually given with vincristine, is one of the front-line treatments for pediatric low-grade gliomas (LGG) (Packer et al., 1993). The combination is associated with a 5-year event-free survival of 39% in sporadic, and 69% in neurofibromatosis type 1 (NF1)-associated LGG (Ater et al., 2016). Other combination regimens, such as thioguanine, procarbazine, lomustine, and vincristine, cisplatin/etoposide, bevacizumab/irinotecan and temozolomide have produced consistent, durable responses (Packer et al., 1997; Massimino et al., 2010) but each of these therapies has drawbacks which include late toxicities such as second malignant neoplasms, infertility, vasculopathy/stroke or toxicity due to concomitant therapies (Ruggiero et al., 2010) as well as concerns that the efficacy of these other options is no better than the first line regimen (Bowers et al., 2006; Eichenauer et al., 2014).

Currently, the combination vincristine/carboplatin is the most widely adopted multi-agent chemotherapy for childhood LGG (Perilongo, 2005). However, one of the most common side effects leading to premature treatment cessation is carboplatin hypersensitivity reaction (CHR). The frequency of CHRs has been reported to be 7–78% while the median number of administered carboplatin doses at the first reaction ranges between 7 and 9 (Lazzareschi et al., 2002; Gnekow et al., 2004).

Management options include cessation of carboplatin and switching to another agent, premedication with antihistamines and/or corticosteroids, and carboplatin desensitization. The reported success rate for completion of chemotherapy in children after various modifications ranges between 0 and 75%, but previous studies were generally either performed on small groups or reported from multiple centers with different management strategies (Broome et al., 1996; Ogle et al., 2002; Gnekow et al., 2004; Lafay-Cousin et al., 2005).

The aim of this study is to summarize the incidence and the clinical features of CHRs occurring in children with LGG treated with carboplatin and vincristine and their impact on treatment efficacy, in order to outline possible adequate prevention and management strategies.

## Patients and methods

### Study population

This retrospective study included 47 children with LGG aged 1–18 years, who were treated with carboplatin and vincristine at the Pediatric Oncology Division of the Catholic University of Rome between January 1994 and July 2015. The median age of the patients was 3.4 years (range, 1–16), 25 males (53.2%), and 22 females (46.8%). Six patients (12.7%) had LGG associated with NF1. The tumors were located in diencephalon in 36 children (76.5%); other locations included the spinal cord, brainstem, cerebral hemisphere and isolated optic nerve (Table 1).

**Table 1:** **Patients' clinical and pathological characteristics**.

**Patients characteristics**	**CHR status No. patients (%)**	
	**CHR positive, *N* = 16**	**CHR negative, *N* = 31**
Median age at diagnosis of LLG months (range)	34 (12–72)	53.5 (12–156)
NF1	2 (12.5)	5 (16.1)
Boys/girls	8/8	14/17
Diencephalic location	13 (81.2)	24 (77.4)
Monthly carboplatin administration	10 (62.5)	8 (25.8)
Weekly carboplatin administration	6 (37.5)	23 (74.2)

Two populations were identified: the first consisting of 29 children, treated from 1994 to 2003 with carboplatin and vincristine according to Packer et al. (1997); the second consisting of 18 children treated from 2004 to 2015 according to protocol SIOP LGG 2004. The population treated according to Packer et al. received a combination of vincristine 1.5 mg/m^2^ and carboplatin 175 mg/m^2^ on a weekly schedule. The induction course consisted of carboplatin at a dose of 175 mg/m^2^/week for 4 consecutive weeks, followed by 2 weeks rest period and again carboplatin for 4 weeks. Vincristine at a dose of 1.5 mg/m^2^ was given by intravenous bolus weekly for 10 weeks concurrently with carboplatin. Maintenance therapy consisted of at least eight cycles. Each cycle included 4 weekly doses of carboplatin at 175 mg/m^2^/week and 3 weekly doses of vincristine at 1.5 mg/m^2^/week (given concomitantly with the first 3 weeks of carboplatin), followed by 2 weeks of rest for a total of 6 weeks. Population treated according to protocol SIOP LGG 2004 received a combination of vincristine 1.5 mg/m^2^ (max 2 mg) and intravenous 1 h infusion of carboplatin at a dose of 550 mg/m^2^. Induction standard consisted in a weekly regimen of vincristine and carboplatin every 3 weeks for 10 weeks. Following the 10 week-induction treatment the combination of vincristine and carboplatin was given three times at 4-weekly intervals. Consolidation was achieved by the continuous, simultaneous application of vincristine and carboplatin. However, treatment was prolonged up to week 81 by extending treatment intervals to 6 weeks, and vincristine was given at a more intense schedule on day 1, 8, and 15 of each cycle. All patients received antiemetic ondansetron before carboplatin infusion. Carboplatin was administered, in both treatments, in 1-h infusions in saline solution. The severity of allergic reactions was evaluated according to the toxicity scale used by the National Cancer Institute^1^(CTCAE, v4.0). Intradermal testing was performed in 15 patients according to the generally applied techniques on the volar surface of the forearm. In particular, the prick test and intradermal skin tests were performed with carboplatin at a concentration of 1 mg/ml; the pacth test was performed utilizing 10 mg/ml of carboplatin.

This retrospective study was approved by the Ethics Committee of the Sacred Heart Catholic University.

### Treatment after CHR

The institutional clinical practice for most patients with a LGG and a carboplatin CHR was to attempt re-exposure. Patients treated with the schedule according to Packer et al., were re-exposed using the desensitization protocol by Lazzareschi et al. (2002) which consists of a standard dose of carboplatin (175 mg/m^2^ in 100 ml saline solution) at an increasing infusion rate as follows: 0.3 mg/m^2^/min for 30 min, 0.6 mg/m^2^/min for 30 min, 1.8 mg/m^2^/min for 30 min, and 2.4 mg/m^2^/min for 30 min (Table 2). Children treated following protocol SIOP LGG 2004 were re-exposed, according to protocol guidelines, using prolonged infusion time (4 h).

**Table 2:** **Carboplatin desensitization protocol**.

**Infusion rate (mg/m^2^/min)**	**Time (min)**
0.3	30
0.6	30
1.2	30
1.8	30
2.4	30

The clinical characteristics evaluated in the study were age, gender, histopathology, and anatomic location of the tumor, presence of NF1, type and time of onset CHR, type of modification in case of CHR, and outcome (continuation or discontinuation of carboplatin therapy after rechallenge).

After the frequency of CHR was described, Fisher's exact test or chi-square analysis were used to assess the potential predicting factors of CHR. Mann-Whitney *U*-test was used to compare the independent groups. Statistical significance was considered as a *P* < 0.005. STATA 14 for Windows program was used for all statistical analyses.

## Results

All 47 patients were eligible for our study. Sixteen (34%) children were identified as having a CHR; 6 (20.7%) occurred in the group treated according to Packer et al., and 10 (55.5%) occurred in the group treated according to protocol SIOP LGG 2004. Age of patients ranged between 12 and 72 months at the time of the reaction to carboplatin. Fifteen children had juvenile pilocytic astrocytoma and one child had fibrillary astrocytoma.

The median number of carboplatin doses administered at the first episode of CHR was 8 (range, 3–11).

CHR occurred earlier during the weekly schedule with a median of 7 infusions (range, 3–9) and with a median cumulative dose of 1,225 mg/m^2^ (range, 525–1,575 mg/m^2^) compared with a median of 8.5 infusions (range, 5–11) and a median cumulative dose of 4,675 mg/m^2^ (range, 2,750–6,050 mg/m^2^) on the monthly schedule. The occurrence of CHR was not related to a specific cumulative dose of carboplatin (*P* > 0.7). The cumulative incidence of CHR appeared to increase with the number of infusions without any plateau effect (*P* < 0.04).

Of the 16 children who developed CHR, the severity of the reaction was grade 1 in 2 patients, grade 2 in 12 patients and grade 3 in 2 patients according to the National Cancer Institute. There were no grade 4 allergic reactions (Table 3). The patients who developed grade 3 bronchospasm were treated with hydrocortisone and adrenalin and symptoms were resolved within 1 h. Chlorphenamine and hydrocortisone were administered intravenously to patients with grade 2 reactions with rapid recovery.

**Table 3:** **Patients' characteristics with CHRs**.

**Patients characteristics**	**Treatment schedule**	
	**Protocol by Packer et al., *N* = 6**	**Protocol SIOP LGG 2004, *N* = 10**
Boys/girls	4/2	4/6
Median age at reaction months (range)	20 (12–60)	39.5 (12–72)
Median No. of infusion (range)	7 (3–9)	8.5 (5–11)
Median cumulative dose (mg/m^2^)	1225 (525–1,575)	4675 (2,750–6,050)
Grade CHR 1-2	5 (83.6)	9 (90)
Grade CHR 3	1 (16.4)	1 (10)

Among the 16 patients who developed CHR, all patients were re-exposed to carboplatin: 6 with desensitization, and 10 prolonging the infusion time according to SIOP LGG 2004 guidelines. The median interval between CHR and rechallenge was 21 days (range, 8–114). There were no significant differences in gender, NF1 status, mean age at treatment initiation, or tumor location between the patients who were re-exposed to carboplatin using a prolonged infusion time or alternatively a desensitization protocol.

There was difference in the success of carboplatin rechallenge between the patients who were treated by prolonging the infusion time only (1 of 10, or 10% successfully rechallenged) compared to those who underwent a desensitization protocol (6 of 6, or 100% successfully rechallenged) (*P* = 0.001).

Of the 10 patients who received post CHR with prolonged infusion time 9 patients discontinued carboplatin because of either recurrent same grade CHR or because of progression to a higher grade CHR (6 and 3 patients, respectively). Patients who failed carboplatin rechallenge received a median of 9 doses at the first reaction (range, 5–11), and a median of 2 doses post rechallenge, at the second reaction (range, 1–8) (Table 4).

**Table 4:** **Rechallenge and outcome in patients with CHR**.

**Patients (n)**	**No. of carboplatin dose until first CHR**	**Grade at first CHR**	**Modification**	**Time to last dose (days)**	**No. of carboplatin dose after modification**	**No. of total courses**	**Grade at second CHR**	**Outcome**
1	9	3	Desensitization	22	–	9	–	Discontinued carboplatin/dead
2	6	2	Desensitization	20	15	21	–	Off-therapy/alive
3	9	2	Desensitization	114	8	17	–	Off-therapy/alive
4	7	2	Desensitization	9	5	12	–	PD, discontinued carboplatin/dead
5	3	2	Desensitization	8	3	6	–	Off-therapy/alive
6	7	2	Desensitization	20	3	10	–	Off-therapy/alive
7	8	2	Prolonged infusion time	21	2	9	2	Swiched to other chemotherapy
8	6	2	Prolonged infusion time	31	8	13	2	Swiched to other chemotherapy
9	11	1	Prolonged infusion time	25	1	11	2	Swiched to other chemotherapy
10	7	1	Prolonged infusion time	21	5	11	2	Swiched to other chemotherapy
11	9	2	Prolonged infusion time	41	2	10	2	Swiched to other chemotherapy
12	5	2	Prolonged infusion time	15	3	7	2	Swiched to other chemotherapy
13	11	2	Prolonged infusion time	29	1	11	2	Swiched to other chemotherapy
14	11	2	Prolonged infusion time	18	2	12	2	Swiched to other chemotherapy
15	9	2	Prolonged infusion time	20	3	11	3	Swiched to other chemotherapy
16	8	3	Prolonged infusion time	22	4	12	–	Off-therapy/alive

Of 47 cases, 5 (10.6%) were deceased, resulting in 10 and 20 years OS of 89 and 82%, respectively. Thirty-seven percent of the population experienced at least 1 progression of the primary tumor, while 17% had multiple tumor progression events. The 10 and 20-year PFS was 61 and 52, 8%, respectively. Among the 7 patients continuing on chemotherapy with carboplatin (after desensitization protocol in 6 patients and prolonging infusion time in 1 patient), there was observed to be: 1 complete response, 2 partial responses, 2 stable disease and 2 progressive disease (20-year OS = 85.7%, and 20-year PFS = 57.2%).

## Discussion

A number of cytotoxic agents are known to cause severe allergic reactions leading to treatment discontinuation. The use of carboplatin in the treatment of children with brain tumors is associated with hypersensitivity reactions. The incidence of allergic reactions varies according to the different schedules of carboplatin, ranging between 7 and 78% (Chang et al., 1995; Schiavetti et al., 1999; Mahoney et al., 2000; Demaerel et al., 2002; Lazzareschi et al., 2002). Yu et al. identified the weekly dosing schedules of carboplatin as a risk factor for allergic reaction in brain tumor patients (Yu et al., 2001). The risk of hypersensitivity appears to increase with repeated exposure to carboplatin, generally after several courses of therapy. This could explain the higher prevalence of allergic reaction recorded in specific patients treated with protocols that use a repeated cycle of carboplatin. In contrast with the experience reported by Yu et al. (2001), we found that patients receiving weekly dosing had an incidence of CHR no greater than patients on the 3 weekly carboplatin protocol. However, reactions occurred 6 months earlier in the weekly dosing group (2.1 vs. 8 months after treatment initiation, respectively). The exact mechanism of carboplatin hypersensitivity is neither adequately known nor exhaustively elucidated (Chiaretti et al., 2004). Hypersensitivity reactions have been reported to all platinum containing chemotherapeutics (Makrilia et al., 2010; Ruggiero et al., 2013b). Various immunological and non-immunological mechanisms can be involved in the pathogenesis of HSRs.

A type IgE mediated hypersensitivity is supported by the rising incidence of hypersensitivity with repeated doses, and the occurrence of positive skin prick test reactions to platinum compounds (Leguy-Seguin et al., 2007).

Most mild reactions seem to caused by alternative mechanism of hypersensitivity non IgE mediated, or rather mast cells and basophil activation with direct release of histamine or cytokine. In support of this mechanism for hypersensitivity is the fact that the reactions often occur midway through infusions of carboplatin rather than at the start, as expected for IgE mediated reactions. In our cohort, reactions occurred within minutes after initiation of the infusion or midway through the infusion or else at the end of infusion or several hours after the drug was delivered. This differs from the typical IgE mediated sensitivity and also intradermal prick tests to carboplatin appear not to be conclusive. There may, in fact, be a combination of the two mechanisms. The lack of sensitivity of skin tests could be accounted for by the low molecular weight of carboplatin which is not immunogenic in its native form.

The results from this retrospective study demonstrate the increased effectiveness of the desensitization protocol compared with the prolonging of the infusion time for continued treatment with carboplatin after an HSR.

Overall, we noted 43.75% of patients were successfully re-exposed to carboplatin (100% for the group re-exposed using a desensitization protocol, and 10% for the group treated by prolonging infusion time), 56% failed the rechallenge. Recently, Lax et al. reported that extended carboplatin infusion does not reduce frequency of hypersensitivity reaction in the retreatment of patients with recurrent platinum-sensitive cancer (Lax et al., 2017).

Moreover, as suggested by various authors, premedication with anti-histamines and/or corticosteroids is able to prevent an allergic reaction only in a limited number of patients with mild or moderate reactions. Moreover, the prolonged use of steroids is associated with long term side effects in children and adolescents, such as mood changes, weight gain and osteoporosis (Ruggiero et al., 2013a). The success of re-exposure in the studies that have used only premedication was between 0 and 28% (Genc et al., 2012).

We used a protocol of desensitization only on the progressive infusion rate with subsequent prolonging of the infusion time of carboplatin without premedication. The use of small starting doses with a progressive increase is thought to consume an IgE antibody and slowly avoid an acute reaction (CTCAE, v4.0). Prolonging infusion time also allows chemotherapy agent antigens to be delivered in a less concentrated fashion per unit of time and therefore decreases antigenic exposure. The protocol was well tolerated and allowed patients to be treated with response to carboplatin.

In our study, an increased severity of CHR on re-exposure was recorded in 3 patients. The increased severity of hypersensitivity re-exposure reactions might subject the patients to the risk of anaphylaxis. Therefore, the benefits of continuing carboplatin chemotherapy should be carefully weighed against the risk of potential hazards.

Some authors have tried to detect predictive factors for CHR (Markman et al., 2003a,b). Currently, there is no accurate predictive factor to identify which patients will truly benefit from altering CHR techniques, and no definitive recommendations can be drawn from our experience. Our results suggest that desensitization protocol is more effective than the extension of the infusion time. The success of re-exposure with desensitization protocol in our study was 100%. Literature data show a success of desensitization between 20 and 75%. This difference could be due to the different patterns of desensitization used. The main variables influencing the success of re-exposure appear to be: the starting dose, the infusion rate and the number of increments (Lafay-Cousin et al., 2005; Ruggiero et al., 2013a; Dodgshun et al., 2016; Shah et al., 2016). A lower starting dose, a slow infusion, and a number of increments greater than or equal to 4, are associated with a greater probability of success.

In conclusion, patients responding to carboplatin chemotherapy might be good candidates for carboplatin chemotherapy even when a CHR occurs. Carboplatin has proven efficacy and a low toxicity profile; termination of the therapy at the first episode of CHR does not seem justified; a desensitization protocol should be considered for patients sensitive to treatment with carboplatin in order to avoid the discontinuation of an effective chemotherapy and improve their chances of cure.

## Ethics statement

This study was carried out in accordance with the recommendations of Ethics Committe of the Sacred Hearth Catholic University of Rome with written informed consent from all subjects. All subjects gave written informed consent in accordance with the Declaration of Helsinki. The protocol was approved by the Ethics Committe of the Sacred Hearth Catholic University of Rome.

## Author contributions

Study conception and design: AR, MC, RR, and DR. Acquisition of data: MC, DR, SM, GA, and PM. Analysis and interpretation of data: AR, GA, RR, MC, GA, and SM. Drafting of manuscript: MC, AR, and DR. Critical revision: AR, MC, DR, and RR.

### Conflict of interest statement

The authors declare that the research was conducted in the absence of any commercial or financial relationships that could be construed as a potential conflict of interest.

## Footnotes

^1^National Cancer institute, Common Terminology Criteria for Adverse Evens v4.0, NCI, NIH, DHHS. May 29, 2009. NIH publication # 09-7473.

## Abbreviations

LGG: low grade gliomas
CHR: carboplatin hypersensitivity reaction
NF1: neurofibromatosis type 1.
